# A new myxozoan parasitizing *Mesonauta festivus* (Cichliformes: Cichlidae) from the lake region in the municipality of Tartarugalzinho, Eastern Amazon, Brazil

**DOI:** 10.1590/S1984-29612024059

**Published:** 2024-10-07

**Authors:** Abthyllane Amaral de Carvalho, Roger Leomar da Silva Ferreira, Lilia Suzane de Oliveira Nascimento, Luize Cristine Pantoja dos Reis, Kalieli Martins Silva, Luana Silva Bittencourt, Marcela Nunes Videira, Elane Guerreiro Giese

**Affiliations:** 1 Programa de Pós-Graduação em Biologia de Agentes Infecciosos e Parasitários, Instituto de Ciências Biológicas, Universidade Federal do Pará – UFPA, Belém, PA, Brasil; 2 Laboratório de Morfofisiologia e Sanidade Animal, Universidade do Estado do Amapá – UEAP, Macapá, AP, Brasil; 3 Programa de Pós-Graduação em Ciências Ambientais, Universidade Federal do Amapá – UNIFAP, Macapá, AP, Brasil; 4 Laboratório de Histologia e Embriologia Animal, Instituto de Produção e Saúde Animal, Universidade Federal Rural da Amazônia – UFRA, Belém, PA, Brasil

**Keywords:** Parasitology, myxozoa, fish, urinary bladder, Parasitologia, myxozoa, peixe, vesícula urinária

## Abstract

The Amazon is the largest river basin in the world and it is home to the greatest diversity of freshwater fish in the world. *Mesonauta festivus* is a cichlid popularly known as flag cichlid, widely distributed throughout South America. The diversity of parasites in fish from the Amazon region is still underestimated, due to the high fishes diversity. The Myxozoa class has a universal distribution, with some specimens being pathogenic to some fish. The aim of this work was to describe a new species of *Hoferellus* in *M. festivus*. The fish were collected in the lake region, municipality of Tartarugalzinho, in the state of Amapá, Brazil. The new species was found parasitizing the urinary bladder of *M. festivus*. Spores were 11.5 ±1.1 (10.4-12.6) µm long and 10.9 ±1 (9.9-11.9) µm wide, and polar capsules were equally sized, measuring 4.9 ±0.5 (4.4-5.4) µm long and 3.4 ±0.9 (2.5-4.3) µm wide, with a pyriform shape, convergent with the apical region of the spore. The polar filament was wound with 5 to 6 turns. Morphological, morphometric, molecular and phylogenetic analysis proved that it is a new species of *Hoferellus* in the Amazon region.

## Resumo

A Amazônia é a maior bacia hidrográfica do mundo e abriga a maior diversidade de peixes em água doce. *Mesonauta festivus* é o ciclídeo conhecido popularmente como acará festivo, com ampla distribuição pela América do Sul. A diversidade de parasitos em peixes da região amazônica ainda é subestimada, devido à sua alta variedade. A classe Myxozoa tem distribuição universal, apresentando alguns exemplares patogênicos a alguns peixes. O objetivo deste trabalho foi descrever uma nova espécie de *Hoferellus* em *M. festivus.* Os peixes foram coletados na região dos lagos, município de Tartarugalzinho, no estado do Amapá, Brasil. A nova espécie foi encontrada parasitando a vesícula urinária de *M. festivus*. Os esporos apresentaram 11.5 ±1.1 (10.4-12.6) µm de comprimento e 10.9 ±1 (9.9-11.9) µm de largura, e cápsulas polares possuíam tamanhos iguais, medindo 4.9 ±0.5 (4.4-5.4) µm de comprimento e 3.4 ±0.9 (2.5-4.3) µm de largura, apresentando formado piriforme, convergente com a região apical do esporo. O filamento polar estava enrolado com 5 a 6 voltas. As análises morfológicas, morfométricas, moleculares e filogenéticas comprovaram que essa espécie encontrada, difere das demais descritas, tratando-se de uma nova espécie de *Hoferellus* na região amazônica.

## Introduction

The Amazon is the largest river basin in the world and is home to the greatest diversity of freshwater fish in the world, with approximately 2500 species, which represents 20% of all freshwater fish worldwide. This basin passes through nine countries in South America, but approximately 65% of its territory is in the northern region of Brazil (Confalonieri et al., 2014; Duponchelle et al., 2021).

Cichlids are the third largest family in terms of number of exclusively freshwater fish species in Brazil and come in various shapes and sizes (Buckup et al., 2007). *Mesonauta festivus* Heckel, 1840 is popularly known as flag cichlid that is widely distributed throughout South America and has high economic value within the aquarium hobby (Pires et al., 2015).

The diversity of parasites in fish from the Amazon region is still underestimated because of the high fishes diversity and high degree of endemism in the region, which favor parasitological diversity (Virgilio et al., 2021). Among the largest groups that parasitize fish in the Neotropical region is the Class Myxozoa (Phylum Cnidaria) Grassé 1970 (Kyger et al., 2021), which has around 2,596 described species distributed in 67 genera (Okamura et al., 2018).

The Class Myxozoa has a universal distribution, with some species being pathogenic to certain species of fish. They have normally microscopic spores between 10 and 50 micrometers approximately, with simple morphology and a complex life cycle that is still little studied (Atkinson et al., 2018). The genus *Hoferellus* Berg, 1898 are generally parasites of freshwater hosts and tend to be coelozoic with intracellular development, which can cause kidney disease in their host, as well as polycystitis, kidney swelling or abdominal distension, as this genus has an affinity for the excretory system of its host (Alama-Bermejo et al., 2016; Pereira et al., 2022).

Two species of myxozoa have already been described for *M. festivus*, *Sphaerospora festivus* Bittencourt et al., 2021 and *Ceratomyxa macapaensis* Bittencourt et al., 2022, collected from specimens of flag cichlid from the Piririm river, in the municipality of Macapá in the state of Amapá. This work described a new species of myxozoan in *M. festivus* from the Lakes Region, in the municipality of Tartarugalzinho, state of Amapá, Brazil, based on phylogenetic, molecular and morphological analyses.

## Material and Methods

### Host collection

Twenty-three specimens of *M. festivus* were collected from the Tartarugalzinho river in the lake region of the municipality of Tartarugalzinho, northern region of the state of Amapá. The specimens were captured by the team from the Amazon Aquatic Organism Health research group (SOAA) and assisted by local fishers using a trawl net with 30 mm between the knots, line and hooks, gill net and cast net, during the period from November 2021 to December 2022.

The specimens were packaged and transported alive in thermal boxes with the help of battery-powered pumps for artificial aeration, to the Morphophysiology and Animal Health Laboratory (LABMORSA) of the State University of Amapá (UEAP), where they were placed in aquariums equipped with electric pumps and filters. Later on parasitological analyses were carried out.

### Morphological analysis and parasite collection

For the analyses, the fish were anesthetized with MS 222 SIGMA and desensitized by spinal section, after which the fish's biometry was performed to obtain weight (g) and length (cm). After biometry, the entire body surface was analyzed using a binocular stereoscopic microscope to check for the presence of lesions, cysts or loss of lining. Subsequently, an incision was made in the ventral region in order to expose the coelomic organs for individual analysis of each segment.

Small fragments were removed from each organ of the fish with the aid of scissors and tweezers and pressed between slides and coverslips where they were analyzed using light microscopy (ML) to confirm the findings. The methodology suggested by Bush et al. (1997) was adopted for calculating prevalence.

In the fragments analyzed where the presence of parasites occurs, the tissues were fixed in Davidson (95% alcohol, formaldehyde, acetic acid and distilled water) and processed using histological techniques for paraffin impregnation, stained using the Ziehl-Neelsen (ZN) technique (Luna, 1968), and later photographed using the MOTICAM 2300 3.0 Pixel attached to a microscope at LABMORSA/UEAP.

For morphometric analysis of fresh myxospores, the recommendations of Lom & Arthur (1989) were followed using n=35. The morphometry of myxospores was expressed as an arithmetic mean in micrometers (µm), followed by standard deviation (SD).

### Molecular and phylogenetic analyses

The cysts and/or tissue fragments parasitized with microparasite spores were collected and fixed in 80% ethyl alcohol at 4°C. The total DNA from each sample collected was extracted using the Wizard® Genomic DNA Purification Kit (according to the manufacturer). DNA samples were quantified by spectrophotometry (Biodro Duo).

The molecular analyses were based on the 18S rDNA sequences, which were amplified using the ERIB1(F) and ERIB10 (R) primers, followed by the MC3 (F) and MC5 (R) primers (NESTED PCR). The final polymerase chain reaction (PCR) volume of 25 µL consisted of 0.2 µL of Taq DNA polymerase (INVITROGEN, Massachusetts, USA), 10 µM of each primer, 3.0 µL of the DNA sample, and 16.8 µL of Master Mix (INVITROGEN, Massachusetts, USA).

Amplification was performed in a MyGene™ Series Peltier thermal cycler (Model MG96G) with the following cycling conditions for the ERIB1 and ERIB10 primers: initial denaturation at 95 ºC for 5 min, followed by 35 cycles of 95 ºC for 30 s, annealing at 56 ºC for 30 s, 72 ºC for 1 min, and final extension at 72 ºC for 10 min. For the second amplification with the MC3 and MC5 primers, the following cycling program was employed: initial l denaturation at 95 ºC for 5 min, followed by 35 cycles of 95 ºC for 30 s, annealing at 55 ºC for 50 s, 72 ºC for 1 min, and final extension at 72 ºC for 10 min. The PCR results were analyzed via electrophoresis on a 1.5% agarose gel in Tris-borate-EDTA buffer. The PCR product was purified using the Illustra™ GFX™ PCR DNA and Gel Band Purification kit, according to the manufacturer's recommendations.

The amplification results were sequenced in the ABI 3730 automatic DNA analyzer using the BigDye v3.1 Cycle Sequencing Ready Reaction Kit (Applied Biosystems) and to confirm the mutations, each sample was sequenced with a forward and reverse primer. The nucleotide sequences obtained were edited and aligned in the BioEdit program (Hall, 2007). Partial sequences were assembled in the Codon Code Aligner software (CodonCode Corporation, Dedham, Massachusetts), and the generated product was compared with sequences deposited in GenBank (Table 1) using the Basic Local Alignment Search Tool (BLASTn) of the National Center for Biotechnology Information (NCBI).

**Table 1:** The genetic distance (*p*) recorded between pairs of *Hoferellus* spp. that comprise the clade of registered *Hoferellus* spp. in Brazilian Amazon and Around the world.

Species	1	2	3	4	5	6	7	8	9
(1) *Hoferellus tartarugualis* n. sp.	-	-	-	-	-	-	-	-	-
(2) *Hoferellus jutubensis*	0.13	-	-	-	-	-	-	-	-
(3) *Hoferellus azevedoi*	0.06*	0.13	-	-	-	-	-	-	-
(4) *Hoferellus gnathonemi*	0.19	0.19	0.18	-	-	-	-	-	-
(5) *Hoferellus gilsoni*	0.13	0.15	0.13	0.13	-	-	-	-	-
(6) *Hoferellus anurae*	0.24	0.26	0.22	0.23	0.20	-	-	-	-
(7) *Hoferellus carassii*	0.21	0.21	0.19	0.23	0.20	0.25	-	-	-
(8) *Hoferellus cyprini*	0.20	0.21	0.18	0.24	0.20	0.24	0.04	-	-
(9) *Hoferellus* sp. ex *C. carpio*	0.20	0.21	0.18	0.24	0.20	0.24	0.04	0.006	-
(10) *Hoferellus alosae*	0.14	0.16	0.13	0.16	0.13	0.23	0.19	0.19	0.19

* The smallest *p* distance found.

To obtain the phylogenetic relationships of each taxon, maximum parsimony and Bayesian analysis (BI) were used, with the help of the programs PAUP 4.0 b10 (Swofford & Sullivan, 2003) and MrBayes 3.1.2 (Ronquist & Huelsenbeck, 2003), respectively. For both analyses, the sequences of organisms related to each taxon that was studied were obtained directly from GeneBank to be used as related and external groups. The BI products were used to build a phylogenetic tree from a set of myxozoan sequences.

## Results

### Morphological description of the spores

**Figure 1:**
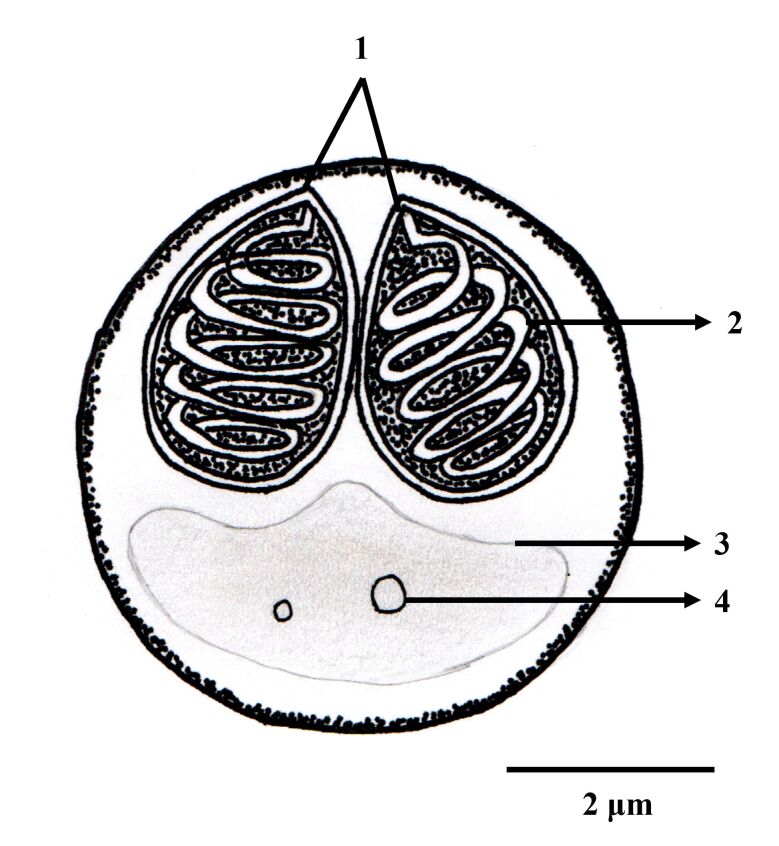
Schematic drawing of the spore frontal view of *Hoferellus tartarugualis* n. sp. 1: Polar capsule; 2: Turns; 3: Sporoplasm; 4: Binucleate cells.

**Figure 2:**
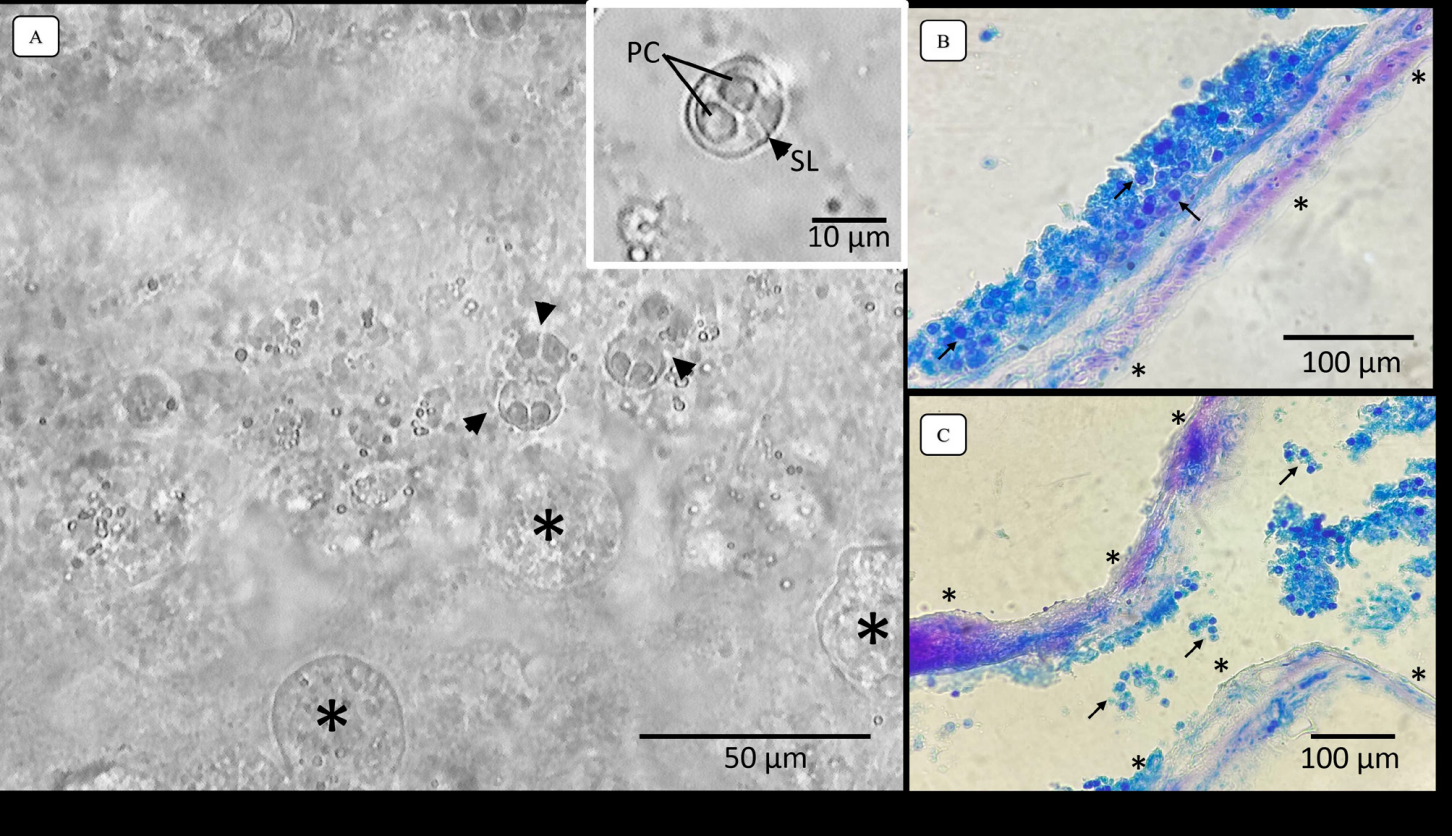
A) Light microscopy of plasmodia (asterisk) of *Hoferellus tartarugualis* n. sp. (arrow head) Scale bar: 50 μm. Highlights: a single spore of *Hoferellus tartarugualis* n. sp., where arrow head indicates suture line (SL). PC: Polar capsules. Scale bar: 10 μm. B) and C) Histological section with Ziehl-Neelsen technique stained of urinary bladder with *Hoferellus tartarugualis* n. sp. (arrow). Asterik indicates the epithelium of urinary bladder. Scale bar: 100 μm.

A total of 23 specimens of *M. festivus* were examined (weight average= 17.3 ± 9.0 g; average length= 7.07 ± 1.09 cm), of which 65% (n=15) were parasitized by *Hoferellus tartarugualis* n. sp. (Figure 1). In the urinary bladder it was possible to observe several dysporic plasmodia of different sizes and shapes (Figure 2A). When outside the plasmodia, the spores were found attached to each other or loose in the liquid (Figure 2A – arrowhead).

Of the fifteen parasitized specimens, 10 were used to obtain morphometric data on spores of *Hoferellus tartarugualis* n. sp. The mature spores were round in shape from sutural view, measuring 11.5 ±1.1 (10.4-12.6) µm in length and 10.9 ±1 (9.9-11.9) µm in width. The suture line and sporoplasm are well defined (Figure 2A - highlighted). On the surface of the spore there were longitudinal lines that extended to the contour of the spore, forming light ridges. The polar capsules have similar sizes, with no significant difference, measuring 4.9 ±0.5 (4.4-5.4) µm in length and 3.4 ±0.9 (2.5-4.3) µm in width, having a pyriform shape, convergent with the apical region of the spore, in towards the suture line. The spiral-shaped polar filament had 5 to 6 turns (Table 2).

**Table 2:** Comparative table of measurements (μm) with standard deviation of *Hoferellus tartarugualis* n. sp. and other *Hoferellus* spp. described. IS: infection site; PCL: polar capsule length; PCW: polar capsule width; PF: number of coils in the polar filament.

Species	Host	Locality	IS	SPORE (μm)			POLAR CAPSULE (μm)			PF	Reference
				Shape	Lenght	Width	Shape	PCL	PCW		
*Hoferellus tartarugualis* n.sp.	*Mesonauta festivus*	Amapá/BR	Urinary bladder	Rounded	11.5 ±1.1 (10.4-12.6)	10.9 ±1 (9.9-11.9)	Pyriform	4.9 ±0.5 (4.4-5.4)	3.4 ±0.9 (2.5-4.3)	5-6	Present study
*Hoferellus azevedoi*	*Chaetobranchus flavescens*	Pará/BR	Urinary bladder	Sub spherical	5.3 ± 0.2 (5.2–5.6)	7.0 ± 0.7 (6.3–7.7)	Pyriform to ovoid	2.5 ± 0.2 (2.3–2.8)	1.8 ± 0.2 (1.6–1.8)	3–4	Matos et al. (2018)
*Hoferellus jutubensis*	*Ageneiosus inermis*	Pará/BR	Urinary bladder	Rounded	6.1 ± 0.2 (5.7–6.3)	5.5 ± 0.3 (5.2–6.0)	Sub spherical	2.5 ± 0.2 (2.3–2.7)	1.7 ± 0.2 (1.4–2.2)	3–4	Pereira et al. (2022)
*Hoferellus gilsoni*	*Anguilla anguila*	Hungary	Urinary bladder	Sub spherical	7.8 (7.2–8.7)	7.6 (6.7–8.7)	Oval	3.5 (2.9–4.3)	2.6 (1.9–3.1)	5–6	Lom & Molnár. (1986)
*Hoferellus anurae*	*Afrixalus dorsalis*	Nigeria	Kidney, intestine, urinary bladder and ureter	Pyramidal or mitra-like	8.0 (7.0–8.9)	7.9 (6.1–7.9)	Pyriform	3.8 (3.2–4.3)	2.0 (1.8–2.1)	6–7	Mutschmann (2004)
*Hoferellus alosae*	*Alosa alosa*	France	Renal tubules	Ellipsoidal	9.1–10.3 (9.7 ± 0.4)	7.7–9.2 (8.4 ± 0.5)	Sub spherical	4.0 ± 0.2 (3.5–4.4)	2.4–3.6 (3.0 ± 0.3)	5	Wünnemann et al. (2016)
*Hoferellus gnathonemi*	*Gnathonemus petersii*	Nigeria	Renal tubules	Rounded	11.9 (10.3–14.3)	11 (9.9–12.7)	Pyriform to ovoid	5.8 (3.7–7.9)	3.7 (2.7–4.8) 3–4	3–4	Alama-Bermejo et al. (2016)
*Hoferellus carassii*	*Carassius auratus*	Russia	kidney, ureter and urinary bladder	Pyramidal or mitra-like	12	6-6.5	Pyriform	4 (2.8–5.8)	2.4 (1.8–3.6)	5–6	Akhmerov (1960)
*Hoferellus cyprini*	*Cyprinus carpio*	France	kidney tubules, ureter and urinary bladder	Slighty round	10–12	6–8	Pyriform	3.1 (2.1–3.9)	2.1 (1.6–2.9)	4–5	Doflein (1898)
*Hoferellus pulvinatus*	*Pangasianodon hypophthalmus*	Thailand	Kidney	Sub spherical	6.5 ± 0.28 (6.0–7.2)	5 ± 0.20 (4.7–5.3)	Drop type	3 ± 0.42 (2.4–3.2)	2.5 ± 0.19 (2.1–2.7)	3	Baska et al. (2009)
*Hoferellus jurachni*	*Alosa tanaica*	Ukraine	Renal tubules, ureteres and urinary bladder.	Rounded to oval	8.5–12.5	6.4–7.5	Spherical	3.1–4.0	2.4–3.2	4	Moshu & Trombitsky (2006)

### Species - Taxonomic Summary

Kingdom Metazoa Linnaeus, 1758

Phylum Cnidaria Hatscheck, 1888

Class Myxozoa Grassé, 1970 (Kyger, 2021)

Subclass Myxosporea Bütschli, 1881

Order Bivalvulida Shulman, 1959

Family Myxobilatidae Shulman, 1953

Genus *Hoferellus* Berg, 1898

Species *Hoferellus tartarugualis* n. sp.

Host: *Mesonauta festivus*

Prevalence: 65% (fifteen specimens)

Site of infection: urinary bladder.

Collection site: Tartarugalzinho river, Tartarugalzinho municipality, state of Amapá, Brazil (coordinates: 01°30’32.5” N; 050°55’10.3 W).

Species deposit: Glass slide with Ziehl-Neelsen stained spores was deposited in the collection of the Amazon Research Institute (INPA), Manaus, Amazonas state, Brazil (accession number: INPA-CND 000099)

GenBank accession number: PP778691

Etymology: The species-specific epithet refers to the municipality in which the fish was collected.

### Phylogenetic and molecular analysis

A partial sequence of 1000 base pairs of the SSU rDNA gene were obtained from the sequencing of spores of *Hoferellus tartarugualis* n. sp. and deposited in GenBank under accession number PP778691. Which comprised G + C (A = 0. 2566, C = 0. 1786, G = 0. 2811, T = 0. 2838). Assuming a GTR + G model of nucleotide substitution, the estimated nucleotide substitution rates were A-C = 1. 4946, A-G = 4. 3420, A-T = 2. 1621, C-G = 0. 7991, C-T = 5. 7664, and G-T = 1.0000, with a gamma distribution (G) of 0. 3260.

**Figure 3:**
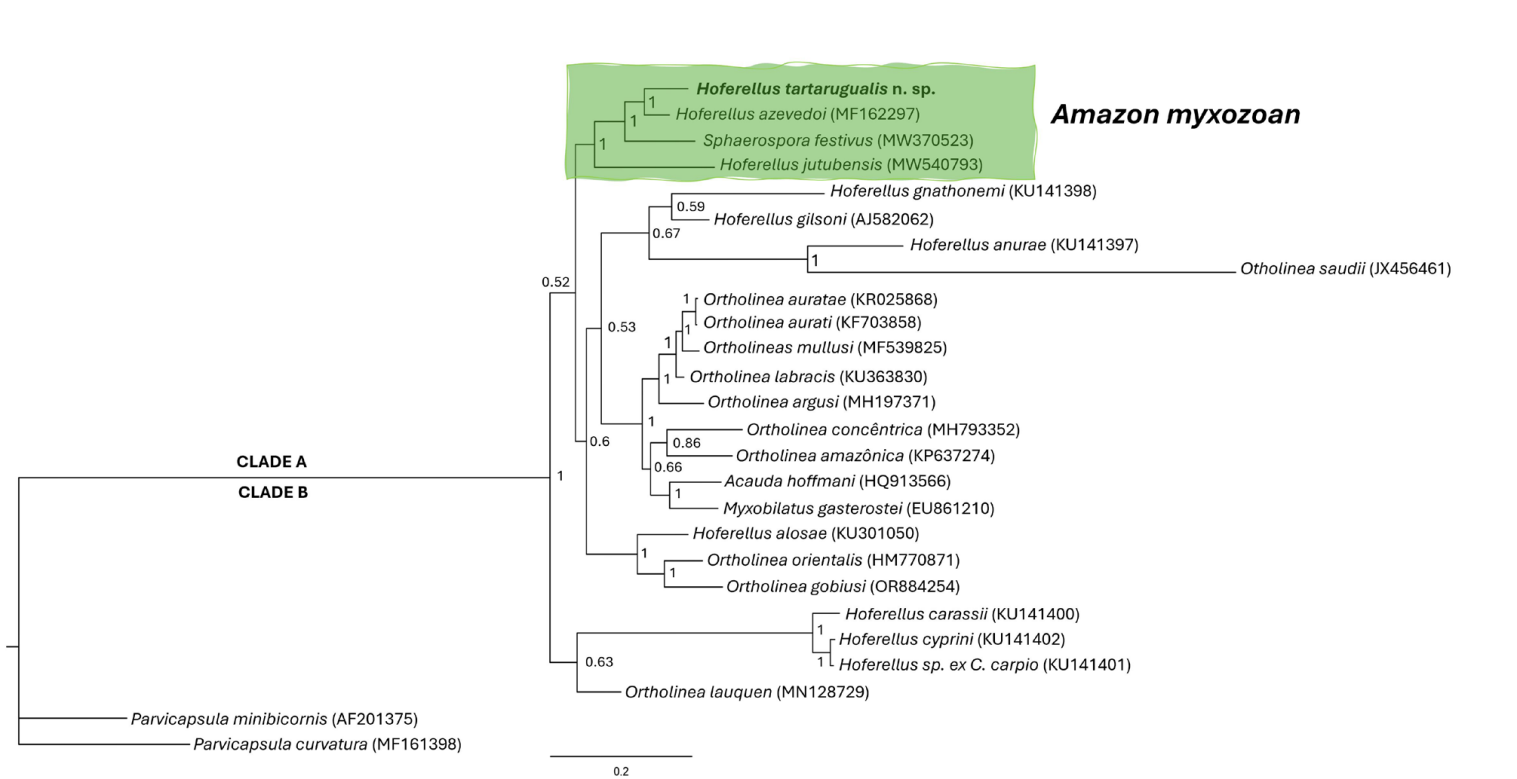
Phylogenetic tree generated by Bayesian inference (IB) through partial alignment of *Hoferellus tartarugualis* n. sp. with SSU r DNA gene sequences of select *Hoferellus, Myxobilatus, Acauda, Sphaerospora* and *Ortholinea* species. Node numbers are indicated for posterior probabilities values calculated by IB

Phylogenetic analysis showed that the genus *Hoferellus* has polyphyletic behavior, with *Hoferellus tartarugualis* n. sp. grouped with myxozoans from the Amazon region (Figure 3), where this new species was found as a sister species to *Hoferellus azevedoi* Matos et al., 2018 (MF162297). In the same subclade, of Brazilian Amazon myxozoans, there was a group with strong nodal support, with *Hoferellus jutubensis* Pereira et al., 2022 (MW540793) and *Sphaerospora festivus* (MW370523), the latter being also recorded in *M. festivus* in the state of Amapá. All myxozoans belonging to the subclade of the Amazon region were described as parasitizing the excretory system of fish.

The smallest genetic distance (*p*) between *Hoferellus tartarugualis* n. sp. and any other species was 6% with *H. azevedoi*, all other distances were greater than 13% (Table 1).

### Remarks

In Brazil, only two species of the genus *Hoferellus* had previously been described, *H. azevedoi* and *H. jutubensis*, with *Hoferellus tartarugualis* n. sp. the third described species of the genus in fish from the Brazilian Amazon. Like the other species of *Hoferellus* in Brazil, *Hoferellus tartarugualis* n. sp. was described parasitizing the urinary bladder of its host. It is worth noting that the new species in this study differs morphologically from the other species discussed above, since *Hoferellus tartarugualis* n. sp. does not present any projection of its valves, unlike *H. azevedoi* (Matos et al., 2018) which presented long projections similar to hairs or filaments at its posterior end, while *H. jutubensis* (Pereira et al., 2022) presented two projections, one from each valve, which had an elongated oval shape.

The plasmodia of *Hoferellus tartarugualis* n. sp. were dysporic, that is, they had only two spores in each plasmodium, unlike *H. jutubensis* and *H. azevedoi*, which had polysporic plasmodia. The plasmodia of *Hoferellus tartarugualis* n. sp. had formed a round shape and were attached to the epithelium of the urinary bladder (Figure 2B and 2C), as observed in *H. azevedoi*, *H. jutubensis* and *Hoferellus gilsoni* Debaisieux 1925. This indicates that all species of *Hoferellus* from the Amazon region have plasmodia fixation in the epithelium of the urinary bladder.

## Discussion

The genus *Hoferellus* is characterized morphologically by having a miter-like or spherical shape, with polar capsules perpendicular to the suture line and open to the anterior pole of the spore, and with filaments generally oblique to the longitudinal axis of the capsule. (Mutschmann, 2004). There is little molecular data on this genus, which was previously classified based only on morphological data, which is why the genus has undergone constant taxonomic reassignment (Alama-Bermejo et al., 2016).

*Hoferellus tartarugualis* n. sp. had a rounded shape, with the presence of striations on the surface of the spores, a common characteristic of the genus that was also observed in *H. azevedoi, H. gilsoni, Hoferellus cyprini* Doflein, 1898, *Hoferellus carassii* Akhmerov, 1960, *Hoferellus anurae* Mutschmann, 2004 and *Hoferellus alosae* Wünnemann et al., 2016.

According to Alama-Bermejo et al. (2016), the apical region of *Hoferellus* myxospores, in valve view, can be miter-shaped or completely rounded. *Hoferellus tartarugualis* n. sp. had a rounded shape and no projections, different from other *Hoferellus* species from the Amazon region, *H. azevedoi* and *H. jutubensis* (Matos et al., 2018; Pereira et al., 2022), that despite having a sub-spherical and rounded shape, respectively, both had projections of the spore body in their posterior region.

Regarding the morphometry of *Hoferellus tartarugualis* n. sp. spores presented larger measurements of both the spore and polar capsules, when compared to the same *Hoferellus* from the Amazon region (Table 1). In terms of the shape of the spore, *Hoferellus tartarugualis* n. sp. was different from *H. azevedoi*, which had a sub-spherical shape (Matos et al., 2018), but it was similar to *H. jutubenis* and *Hoferellus gnathonemi* Alama-Bermejo et al., 2016 which also have a rounded shape (Pereira et al., 2022; Alama-Bermejo et al., 2016).

The polar capsules of *Hoferellus tartarugualis* n. sp. were smaller in length (4.9 ±0.5 µm (4.4-5.4)) only when compared to the polar capsules of *H. gnathonemi* (5.8 µm (3.7–7.9)). However, none of the lengths were similar to *Hoferellus tartarugualis* n. sp. Regarding the shape of the polar capsules, *Hoferellus tartarugualis* n. sp. has a pyriform shape, just like *Hoferellus anurae* Mutschmann, 2004, *Hoferellus carassii* Akhmerov, 1960 and *Hoferellus cyprini* Doflein, 1898. In terms of the number of turns of the polar filament, *Hoferellus tartarugualis* n. sp. has more turns than *H. azevedoi* and *H. jutubensis*, but the same number as *Hoferellus gilsoni* (Lom et al., 1986) and *H. carassii* (Table 1).

This genus has tropism with the excretory system of the host species and is mostly found in the urinary bladder of the hosts. (Lom & Dyková, 2006). However, species of *Hoferellus* described on the European continent were found parasitizing other organs of the excretory system in addition to the urinary bladder, such as the renal tubules, ureters and kidneys of their host fish, e.g., *H. cyprini* parasitizing *Cyprinus carpio* Linnaeus, 1758, *H. carassii* parasitizing *Cyprinus auratus* Linnaeus, 1758, *H. alosae* parasitizing *Alosa alosa* Linnaeus, 1758 and *Hoferellus jurachni* parasitizing *Alosa tanaica* (Akhmerov, 1960; Alama-Bermejo et al., 2016; Doflein, 1898; Wünnemann et al., 2016; Moshu & Trombitsky, 2006).

Morphological and morphometric descriptions serve as support for the description of new species; however, SSU rDNA sequences provide more reliable criteria for the identification of myxozoans and their respective phylogenetic relationships (Fiala et al., 2015; Matos et al., 2018). Molecular analysis of the genus *Hoferellus* is more complicated due to the small number of SSU rDNA sequences available in Genbank.

In the subclade of Amazonian myxozoans, *Hoferellus tartarugualis* n. sp. was grouped with *H. azevedoi*, *H. jutubensis* and *S. festivus*, with the latter myxozoan also described for *M. festivus.* As proposed by Alama-Bermejo et al. (2016), the genus *Hoferellus* is a typically polyphyletic group, and therefore it is possible to observe that in clade A, where *Hoferellus tartarugualis* n. sp was grouped, there are species of the genus *Hoferellus* and the genus *Ortholinea*, *Myxobilatus*, *Sphaerospora* and *Acauda*, thus demonstrating the polyphyly of the group.

According to the phylogenetic approach, it is proposed that the genus *Hoferellus* be divided into two groups, the *stricto sensu*, composed of *Hoferellus* spp. of cyprinid hosts, and the *lato sensu* group. The phylogenetic analysis of this study demonstrated that *Hoferellus tartarugualis* n. sp. belongs to the *lato sensu* group, whose members have rounded or sub-spherical myxospores, generally found in pairs parasitizing teleost fish. They include *H. azevedoi, H. jutubensis, H. gilsoni* and *H. ganthonemi.*

*Hoferellus tartarugualis* n. sp. had as its sister species *H. azevedoi*, both of which use fish from the Cichlidae family as hosts, which may justify this phylogenetic grouping. This has been suggested by Ferguson et al. (2008), Naldoni et al. (2011) and Adriano et al. (2012), who claim that myxozoans tend to group together following their host's family. The new species in this study grouped with *H. azevedoi* from the Amazon region are all members that belong to the clade called “Freshwater Urinary Bladder” (FW-UB) defined by Fiala et al. (2015).

For *M. festivus*, Bittencourt et al. (2021) and Bittencourt et al. (2022) described two species of myxozoans, *S. festivus* and *Ceratomyxa macapaensis*, parasitizing the urinary bladder and gallbladder, respectively. Therefore, *Hoferellus tartarugualis* n. sp. is the third myxozoan species described for *M. festivus*, and it is worth noting that all myxozoan species described for this host were recorded in the state of Amapá.

## Conclusions

Based on morphological and morphometric characteristics and phylogenetic analyses, we can state that *Hoferellus tartarugualis* n. sp. stands out when compared with the other species of existing *Hoferellus*, thus confirming that it is a new species of myxozoan. This is the second description of parasites of the Class Myxozoa for the municipality of Tartarugalzinho and the third species of *Hoferellus* described in the Brazilian Amazon region. Furthermore, this description contributes to a better knowledge of the diversity of microparasites in the Amazon region and provides an important advance in understanding the morphology, distribution, biodiversity and evolution of this group.
